# Transfer RNAs-derived small RNAs and their application potential in multiple diseases

**DOI:** 10.3389/fcell.2022.954431

**Published:** 2022-08-22

**Authors:** Xiaohua Chu, Chenyang He, Bo Sang, Chaofei Yang, Chong Yin, Mili Ji, Airong Qian, Ye Tian

**Affiliations:** ^1^ Lab for Bone Metabolism, Xi’an Key Laboratory of Special Medicine and Health Engineering, Key Lab for Space Biosciences and Biotechnology, Research Center for Special Medicine and Health Systems Engineering, NPU-UAB Joint Laboratory for Bone Metabolism, School of Life Sciences, Northwestern Polytechnical University, Xi’an, SN, China; ^2^ Department of Oncology, The Second Affiliated Hospital of Xi’an Jiaotong University, Xi’an, China; ^3^ Department of Clinical Laboratory, Academician (expert) Workstation, Lab of Epigenetics and RNA Therapy, Affiliated Hospital of North Sichuan Medical College, Nanchong, China

**Keywords:** tRNA, tRNA-derived small RNAs (tsRNA), non-coding RNA, mechanism, diseases

## Abstract

The role of tRNAs is best known as adapter components of translational machinery. According to the central dogma of molecular biology, DNA is transcribed to RNA and in turn is translated into proteins, in which tRNA outstands by its role of the cellular courier. Recent studies have led to the revision of the canonical function of transfer RNAs (tRNAs), which indicates that tRNAs also serve as a source for short non-coding RNAs called tRNA-derived small RNAs (tsRNAs). tsRNAs play key roles in cellular processes by modulating complicated regulatory networks beyond translation and are widely involved in multiple diseases. Herein, the biogenesis and classification of tsRNAs were firstly clarified. tsRNAs are generated from pre-tRNAs or mature tRNAs and are classified into tRNA-derived fragments (tRFs) and tRNA halves (tiRNA). The tRFs include five types according to the incision loci: tRF-1, tRF-2, tRF-3, tRF-5 and i-tRF which contain 3′ tiRNA and 5′ tiRNA. The functions of tsRNAs and their regulation mechanisms involved in disease processes are systematically summarized as well. The mechanisms can elaborate on the specific regulation of tsRNAs. In conclusion, the current research suggests that tsRNAs are promising targets for modulating pathological processes, such as breast cancer, ischemic stroke, respiratory syncytial virus, osteoporosis and so on, and maintain vital clinical implications in diagnosis and therapeutics of various diseases.

## 1 Introduction of tRNA-derived small RNAs

Transfer RNAs (tRNAs) are an essential type of RNA involved in protein biosynthesis (Fernández-Millán et al., 2016). tRNAs can recognize the codons in mRNA by their anticodons and transfer the amino acids which correspond to the codon to the ribosome accurately (Bastide et al., 2018). Mature tRNAs can be processed from precursor tRNAs (pre-tRNAs) by RNA polymerase Ⅲ (Bonhoure et al., 2020).

With the development of high-throughput sequencing technology, tRNA was found to be the source of a class of small noncoding RNAs (ncRNAs) with important functions (Anderson et al., 2016). These ncRNAs are called tRNA derived small RNAs (tsRNAs) which are specific conserved molecules produced by accurate regulation instead of random tRNA degradation products (Kumar et al., 2014), and can be mainly divided into tRNA derived fragments (tRFs) and tRNA halves (tiRNA) according to the cleavage sites (Lu et al., 2021). tRFs, a total of 10,158 kinds, are 14 to 32-base-long RNAs of high abundance that have been characterized from bacteria to humans (Gu et al., 2020). The tRFs are named and classified based on the cleavage positions on pre- and mature tRNAs in various cell types and organisms, such as tRF-1, tRF-2, tRF-3, tRF-5, and i-tRF (Sun et al., 2018). tiRNAs, 29–50 nt in length, are the 5′-and 3′-tRNA halves generated by angiogenin (ANG) in the anticodons of mature tiRNA under various stress conditions (Akiyama et al., 2021). Meanwhile, angiogenin-independent cleavage also exists (Rashad et al., 2021).

Recent studies identified that novel tsRNAs play important roles both in transcriptional and posttranscriptional gene regulation (Garcia-Silva et al., 2014). tsRNAs engage in multiple molecular mechanisms such as RNA modification, gene regulation, protein synthesis, and various fundamental cellular functions (e.g., cell proliferation and stress response) (Shen et al., 2018). In addition, aberrant expression of tsRNAs is found in different diseases such as pathological stress injuries, multiple types of cancers, virus infection, and neurodegenerative diseases (Kim et al., 2020). For example, the heterogeneity and stability of tsRNAs allow them to be suitable biomarkers for cancer diagnosis and prognosis, moreover, some tsRNAs can alleviate the dilemma of acquiring drug resistance (Wang et al., 2019). To further investigate the functions of tsRNAs, more techniques are urgently needed such as Deep sequencing and Microarray (Wang et al., 2020b). Also, databases of tsRNAs help a lot to obtain the information of tsRNAs effectively (Wang et al., 2020b). In this review, the biogenesis and discovery of tsRNAs were expounded as well as the major biological functions of tsRNAs, recent studies on the roles of tsRNAs in various diseases were also delineated here (Table 1). Besides, the clinical application potential of tsRNAs was discussed.

**TABLE 1:** Roles of functional tsRNAs in different types of cancers.

Cancer	tsRNA	Type	Function/Target gene	References
Breast cancer	tRF-2 derived from tRNA-Glu, tRNA-Asp, tRNA-Gly, and tRNA-Tyr	tRF-2	Bind to YBX1 by replacing 3′UTR and suppress cancer cell proliferation and metastasis	Cui et al., (2018); Goodarzi et al., (2015)
	tRF-Glu-YTC (Y can be C or T) Asp-GTC	i-tRF	Inhibit cancer cell proliferation and metastasis	Falconi et al. (2019); Goodarzi et al., (2015)
	tRF-Gly-TCC			
	tRF-Ser-GCT	i-tRF	Unknown	(M. Falconi, M. Giangrossi, and M. Zabaleta et al., 2019)
Ovarian cancer	a 5′ fragment of tRNA-Glu-CTC	tRF-5	Inhibits cell proliferation	K Zhou et al., (2017a)
	tRF-03357	tRF-5	Promotes cell proliferation, migration, and invasion	Zhang et al., (2019a)
Osteoporosis	tRF-25, tRF-38 and tRF-18	tRF-3, i-tRF	Upregulated in Osteoporosis patients compared with healthy counterpart	Zhang et al. (2018)
Knee Osteoarthritis	tRNA-Cys-GCA	tRF-3	This tRF was able to suppress JAK3 kinase, which resulted in decreased expression of IL-6	Zacharjasz et al. (2021)
Prostate Cancer	tRF-315	tRF-3	tRF-315 targeted the tumor suppressor gene GADD45A, thus regulating the cell cycle	Guerne et al. (1990)
	tRF-544	tRF-5	High expression ratio of tRF-315/tRF-544 indicates poor progression-free survival (PFS)	
	tRF-1001	tRF-1	tRF-1001 is required for cell proliferation	Bowles et al. (2014)
Chronic lymphocytic leukemia	ts-46, ts-47	tRF-1	ts-46 inhibited S1P/ceramide pathways to regulate cell proliferation, and the knockdown of ts-46 promoted integrin-linked kinase (ILK) signaling, which induced cell progression	Pekarsky et al. (2016)
	ts-101, ts-53	tRF-1	ts-53 and ts-101 were often found to be mutated in both CLL and lung cancer, and the mutation of ts-101 in CLL was within the region complementary to the Zeta-chain-associated protein kinase 70 (ZAP-70) promoter	Balatti et al. (2022)
Lung cancer	tRF-Leu-CAG	tiRNA-5	abundantly expressed in NSCLC and to cause GO/G1 cell cycle progression, thus promoting cancer cell proliferation	Shao et al. (2017)
	ts-101, ts-53	tRF-1	Associate with PiwiL2, a vital protein involved in silencing of transposons	Balatti et al. (2017)
	ts-46, ts-47		Inhibit the ability of lung cancer cells to form colonies	
Colorectal cancer	tRF/miR-1280	tRNA-Leu and pre-miRNA	Target ligand JAG2 of Notch signaling, while the Notch signaling pathways were related to cancer cell proliferation, metastasis, and cancer stem-like cells (CSC); tRF/miR1280 suppressed colorectal cancer progression by repressing the Notch signaling	Huang et al. (2017)
	tRF-Gln-CTG	tRF-5c	Negative regulation of c-jun N-terminal kinase (JNK) cascade is enriched in tRF-Gln-CTG. Suppression of JNK cascade can reduce the migration potential of cancer cells *in vitro*	Wang et al. (2020c)
	tRF-Leu-TAG	tRF-5a	In tRF-Leu-TAG, the function of mesenchymal-to-epithelial transition is enriched	

## 2 Origin of tRNA-derived small RNAs

tRNAs consist of a D-loop, T-loop, variable loop and the anticodon loop (Sun et al., 2018). The biogenesis of tsRNAs is the cleavage of either pre-tRNA or mature tRNAs by a variety of specific endoribonucleases including angiogenin (ANG), RNA enzyme Z (RNase Z), and Dicer (Yamasaki et al., 2009). Recently, RNase1 has been indicated to involve the degradation of 1,342 tsRNAs in response to H_2_O_2_ treatment, though it has little relation with biogenesis of tsRNAs (Li et al., 2022b). Based on the different cleaving region within pre-/mature tRNA, tsRNAs can be classified into tRFs and tiRNAs (Zuo et al., 2021).

**FIGURE 1:**
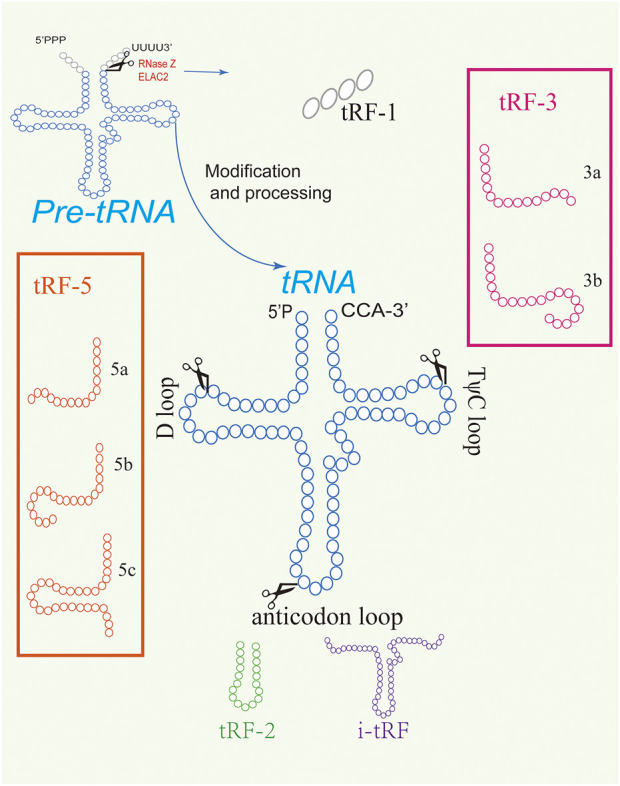
Different types of tRFs. tRFs are derived from the cleavage of precursor or mature tRNAs.

The three main types of tRFs, including tRF-5, tRF-3, and tRF-1, are originated in different cleaving ways (Figure 1). tRF-5, from 5′ end of tRNA, is produced by Dicer cutting at different positions between the D ring and the anticodon ring (Hu et al., 2022). Due to the different cutting sites, tRF-5 can be further divided into tRF-5a (shortest), tRF-5b, and tRF-5c (longest) (Kazimierczyk et al., 2022). tRF-3 is cleaved from the 3′end of tRNA by ANG and Dicer and is divided into tRF-3a and tRF-3b according to the different cutting sites in T ring (Su et al., 2019). tRF-1 is derived from the 3′ untranslated region (3′ UTR) of precursor tRNA, also known as 3′ U-tRF, which is cleaved by RNaseZ with PolyU sequence (Jia et al., 2020). Besides, there are two types of tRFs including tRF-2 and i-tRF which are not as abundant as the other types (Zhu et al., 2019a). tRF-2 is produced from the anticodon loop in hypoxia (Yu et al., 2021). The fragments called i-tRF are from the inner region of mature tRNA and are divided into A-tRF, V-tRF and D-tRF according to cleavage sites in the anticodon loop, variable area, or D loop respectively (Wang et al., 2020c).

tRNA halves (tiRNAs, also called tRNA-derived stress-induced RNAs) were reported to be generated by ANG at the anticodon loop of mature tRNA under stress induction (e.g., arsenite, ultraviolet irradiation, hypoxia, oxidation, amino acid deficiency, starvation, viral infection and so on), namely 5′ tiRNA (the 5′ half of tRNA) and 3′ tiRNA (the 3′ half of tRNA) (Wang et al., 2020b). tiRNA generation can be regulated by tRNA methylation including 1-methyladenosine modification (m1A) and 5-methylcytidine modification (m5C) (Rashad et al., 2020; Liu et al., 2016; Tuorto et al., 2012). It was newly found that ANG was not the only RNase that generated tiRNAs because knocking-out ANG suggested the majority of tiRNAs remained unchanged, except for the 5′ half from tRNA-His-GTG and the 3′ half from tRNA-Asp-GTC (Su et al., 2019). Rashad and his colleagues reported that the generation of certain tiRNAs was not restricted to anticodon site (Rashad et al., 2021). Such non-canonical cleavage takes place *in vivo* mainly in mitochondrial tRNAs, which *in vitro* also produced multiple large-sized tiRNAs depending on the cutting sites yet was not limited to mitochondrial but also cytoplasmic tRNA transcripts (Rashad et al., 2021).Sanadgol et al. (2022) recently confirmed that medium collected from cells growing in stable conditions contained various ex-tsRNAs which were just slightly shorter than arsenite-induced tsRNAs, indicating tsRNAs that represented the acute phase of cells should be distinguished from ex-tsRNAs, because the latter might be derived from cellular repair processes (Sanadgol et al., 2022). Further studies about the biogenesis of tsRNAs are needed to bring new insights into this new category of noncoding RNAs.

## 3 Technology applied in tRNA-derived small RNAs researches

The development and application of deep-sequencing and microarray facilitate the research on tsRNAs (Li et al., 2020). Microarray is a robust, reliable and high throughput approach to analyze the tsRNA expression patterns for a large number of samples (Grelet et al., 2017). The technique begins with extraction of RNA from the samples and the construction of cDNA library from total RNA. Then fluorescently labeled predesigned probes are used to hybridize complementary sequences in the microarray following washing and screening to quantify the images and analyze the expression profiles (Emetu et al., 2018). Deep-sequencing is developed to transcriptome profiling which broadens the view of range and complexity of tsRNAs knowledge (Chen et al., 2017). The method is performed in the following steps: isolating total RNAs, using reverse transcriptase, and preparing a cDNA library (Chai et al., 2021). Every cDNA strand is ligated with adaptors and these ligated fragments is amplified and purified subsequently (Chai et al., 2021). Finally, the sequencing of cDNA is performed using a NGS method (Wang et al., 2020a).

Since tsRNAs are cleavage products from tRNAs which are extensively modified, tsRNAs which contain nucleoside modifications are likely to escape sequencing-based detection, because many tRNA modifications cause pauses or stop during reverse transcription (RT) which is a vital step in most RNA-seq protocols (Motorin et al., 2007). AQRNA-seq and ARM-seq were developed to provide accurate quantification of tsRNA and some other small RNAs. In the AQRNA-seq workflow, adapter begins at the 3′ end, with two randomized nucleotides at the 5′ end of linker 1 to improve T4 ligase efficiency (Jayaprakash et al., 2011). Although not essential for capturing all RNA sequences, ligated RNA then can be treated with Alkane monooxygenase (AlkB) to reduce the levels of RT-blocking methyl modification and excess linker 1 is removed (Dai et al., 2017). A DNA primer complementary to linker 1 is used to accomplish RT and the resulting complementary DNA is 3′-ligated to a custom DNA adapter (linker 2) using T4 DNA ligase (Hu et al., 2021). Excess linker 2 is removed, and PCR amplification is performed for subsequent sequencing (Hu et al., 2021). In ARM-seq, RNA is treated with a dealkylating enzyme called *E. coli* AlkB before RT in library preparation (Cozen et al., 2015). RNAs can be identified efficiently by differential abundance analysis after demethylation (Cozen et al., 2015).

tRNA modifications are important in the regulation of tRNA cleavage and fragment generation, sequencing methods that can detect and quantify tRNA modifications may be helpful for researches in tsRNAs (Anderson et al., 2016). Zhang et al. (2022) have reviewed the recent advances in tRNA-seq methods and divided them into three categories which were RT-misincorporation signature, chemical treatment for specific modification and library construction strategy (Zhang et al., 2022). Methylations are abundant in tRNAs and lead to misincorporation during RT which is one of causes to RT signatures. While certain sequencing techniques take advantages of RT signatures derived from modifications. For example, DM-tRNA-seq and ARM-seq both use *E. coli* AlkB enzyme to pinpoint RT signatures caused by methylation, thus more accurate tRNA sequencing data and the influence of methylations to tRNA fragmentation can be investigated (Zheng et al., 2015; Cozen et al., 2015). Another approach in detecting and quantifying specific modifications by sequencing utilizes chemical treatments to identify selected modification groups through certain chemical reactions. E.g., DM-ψ-seq combines demethylase treatment and carbodiimide reaction that enhances the data quality for ψanalysis in tRNA significantly (Song et al., 2020). For library construction strategy, an optimized solution to acquire sequencing results with high quality of tRNA is construction of adapters that selectively recognize tRNAs in the input samples therefore the portion of sequencing data for tRNA increased. YAMAT-seq utilizes a Y-shaped adapter to specifically ligate mature tRNA mediated by T4 RNA ligase 2, thus assuring the high selectivity for full-length mature tRNA (Shigematsu et al., 2017). AQRNA-seq, using a two-step adapter ligation strategy with an optional AlkB treatment, further addresses potential ligation biases toward specific tRNA molecules in the library preparation process (J. Hu et al., 2021). Hence, these sequencing methods provide more comprehensive and precise measurements of tRNA sequences as well as modifications, and promote the elucidation of biological roles of tsRNAs.

The development of techniques for identifying tsRNAs has boosted the establishment of several tsRNA-related databases (Zuo et al., 2021). Initially, tRF database (tRFdb) is only attempted to present the tRF sequences and read counts from eight species and is available at http://genome.bioch.virginia.edu/trfdb/(Kumar et al., 2015). Later, MINTbase, containing information about sequence, expression abundance, parental tRNAs, and other genomic information, is developed to tabulate tRF information from nucleic and mitochondrial tRNAs and freely available at http://cm.jefferson.edu/MINTbase/(Pliatsika et al., 2016). Then the tRF2Cancer (https://rna.sysu.edu.cn/tRFfinder/statistics.php), a web server applied for identifying tRFs from small RNA sequencing datasets from various cancer types, is created (Zheng et al., 2016). Moreover, OncotRF (http://bioinformatics.zju.edu.cn/OncotRF) provides an integrated view of dysregulated tRFs among cancers (Yao et al., 2020). TsRFun, as a valuable source of cancer-related tsRNAs, evaluates the expression of tsRNAs in 32 cancers, furthermore, CLIP sequencing is utilized to help display the biological functions and the database is available at http://rna.sysu.edu.cn/tsRFun/(Wang et al., 2022) (Table 2). TsRBase (http://www.tsrbase.org) was newly developed to integrate over 14,000 public small RNA-seq data across 20 organisms and to identify protein-binding tsRNAs and their targets for the first time. Such databases serve as a starting point for users to study gene regulation and functional roles of tsRNAs in cancers, thus facilitating the future investigations of the novel class of tsRNAs. Due to complicated origin of tsRNAs, the naming of them is incomplete in current tsRNA databases. Thus, unifying naming standard and integrating annotation information will be helpful for further exploration.

**TABLE 2:** TsRNA databases.

Database	URL link	Application
tRFdb	http://genome.bioch.virginia.edu/trfdb/	A comprehensive database of tRFs by high-throughput sequencing from eight species, including human
MINTbase	http://cm.jefferson.edu/MINTbase/	A repository of tRFs that arise from the nucleic and mitochondrial tRNAs
tRF2Cancer	https://rna.sysu.edu.cn/tRFfinder/statistics.php	A web server to detect tRFs and their expression in multiple cancers
OncotRF	http://bioinformatics.zju.edu.cn/OncotRF	Provide valuable information to identify diagnostic and prognostic biomarkers, develop cancer therapy and study cancer pathogenesis
tsRFun	http://rna.sysu.edu.cn/tsRFun/	Evaluate the expression and prognostic value of tsRNAs in 32 cancers

## 4 Roles of tRNA-derived small RNAs in different diseases

Overall, tsRNAs are involved in multiple kinds of diseases including cancers, neurological disorders, viral infections, and other diseases (Anderson et al., 2016). Some tsRNAs can be prognostic biomarkers because of their elevated expression in patients (Tang et al., 2021). Such overexpressed tsRNAs can be knocked down by specific siRNAs which are regarded as therapeutic targets (Wang et al., 2022b). Some other tsRNAs are downregulated in patients, so the restoration of their intracellular levels may inhibit the disease progression (Xiao et al., 2022). Furthermore, tsRNAs play an important role by binding to 3′ UTR of certain mRNA and inhibit development of diseases (Wang et al., 2020b).

### 4.1 Roles of tRNA derived fragments in diseases

#### 4.1.1 Cancers

##### 4.1.1.1 Breast cancer

Nearly 1.7 million patients are suffering from breast cancer (BC) globally, which has surpassed lung cancer as the most common cancer (Fornier et al., 2012; Sharma, 2019). It has been reported that a few tsRNAs are differentially expressed in BC (Sung et al., 2021). The role of tsRNAs in BC recently has raised attention in scientific research and the number of tsRNAs studied in BC ranks first (Sung et al., 2021).

As reported, the expressions of some tsRNAs are elevated in the subtypes of BC, for example, tRNA-Val-CAC, tRNA-Val-ACC, tRNA-Gly-GCC, tRNA-Gly-CCC, tRNA-Glu-CUC, tRNA-Lys-CUU, and tRNA-His-GUG were highly expressed in 26 triple-negative breast cancer (TNBC) cells (Zhang et al., 2021). Also, overexpression of tRNAi-Met-CAU was found in the BC-associated fibroblasts obtained from patients (Clarke et al., 2016). Probably, these tsRNAs are potential candidates for BC diagnostic, prognostic biomarkers and therapeutic targets.

Some tsRNAs can play anti-tumor role which are usually downregulated in BC patients. For example, tRF3E is a tRF-3 derived from tRNA-Glu-TTC, known as a tumor suppressor (Falconi et al., 2019). The serum tRF3E decreased in patients suffering from HER2-positive BC as the malignancy increased (Falconi et al., 2019). tRF3E brought about the release of p53 mRNA and further promoted p53 translation by competitively interacting with nucleolin (NCL), leading to the inhibition of cancer cell proliferation (Falconi et al., 2019). A novel class of tsRNAs were induced by hypoxic stress (Goodarzi et al., 2015). This class of tsRNAs which were generated from tRNA-Glu, tRNA-Gly, tRNA-Asp and tRNA-Tyr could suppress the development of BC metastasis by interacting with the oncogenic RNA-binding protein (RBP) Y box binding protein 1 (YBX1) and displacing 3′ UTRs of multiple oncogenic transcripts including high-mobility group AT-hook1 (HMGA1), cluster of differentiation 151 (CD151), cluster of differentiation 97 (CD97), tissue inhibitor of metalloproteinase-3 (TIMP3) and protein kinase B (AKT1) from YBX1 resulting in destabilization of these transcripts in BC cells (Goodarzi et al., 2015). YBX1 was expressed in diverse cancers and stabilized various oncogenic transcripts (Li et al., 2022a). Such clues inspired to investigate the roles of tsRNAs overexpressed under hypoxic conditions in suppression of BC metastasis. However, in highly-metastasis BC cells, the tsRNAs mentioned above were not over expressed, which implied that there was a mechanism of attenuating induction of these tsRNAs to evade the tsRNA-mediated modulation of cancer metastasis (Kwon et al., 2021).

Contrarily, certain tsRNAs that are upregulated in BC patients may promote tumor progression. tDR-0009 (tRF-5 derived from tRNA-Gly-GCC-1-1) and tDR-7336 (tRF-5 derived from tRNA-Gly-GCC-1-2) were significantly raised under hypoxia conditions and were reported to enhance the chemoresistance in TNBC which was resulted from the activation of STAT3 phosphorylation (Cui et al., 2018). Such investigation indicated that hypoxia-induced specific tsRNAs might function as tumor suppressors or regulatory factors. While, some tsRNAs with increased levels are beneficial for BC patients. For example, tRFdb-5024a and 5p_tRNA-Leu-CAA-4-1 are positively related to overall survival of BC suffered patients (Shan et al., 2020; Weidenfeld et al., 2018). Higher level of tRFdb-5024a and 5p_tRNA-Leu-CAA-4-1 lead to the decreased risk of death (Shan et al., 2020). The negatively related genes with tRFdb-5024a such as TGF-beta1 and TGF-beta3 are enriched in the EMT pathway and extracellular matrix-regulated proliferation related pathway (Xu et al., 2021). EMT is regarded as a hallmark of human cancer by acquiring cancer stem cell-like traits which accelerates cancer progression and metastasis (Weidenfeld et al., 2018). Extracellular matrix is a major structural component of the tumor microenvironment and affects the recruitment of immune cells, resulting in impairing proliferation and activation of T cells and inducing EMT (Nallanthighal et al., 2019).

Sex hormones and their receptors are critical in the development and progression of the breast and prostate cancers (Hayes et al., 2022). BC progression can be promoted by sustained exposure of estrogen and at least 70% of BCs are identified as estrogen receptor (ER)-positive luminal type whose development and growth are dependent on the estrogen-activated ERα (Whitworth et al., 2022). In prostate cancer, tumorigenesis is promoted by androgen through androgen receptor (AR)-mediated gene-expression (Nanda et al., 2020). It is reported that a series of tsRNAs are responsive to sex hormones and are elevated in ER-positive BC and AR-positive prostate cancer, designating these tsRNAs as sex-hormone-dependent tRNA-derived RNAs (SHOT-RNAs) which are cleaved by ANG, forming 5′-SHOT-RNAs and 3′-SHOT-RNAs (Honda et al., 2015). When ER or AR was knocked down in BC cell lines (MCF-7) or prostate cancer cell lines (LNCaP-FGC), the amounts of 5′-tRNA-Asp-GUC and 5′-tRNA-His-GUG were reduced (Shigematsu et al., 2017). SHOT RNAs are required for cell proliferation. For example, by transfecting siRNA targeting 5′-SHOT-Asp-GUC and 5′-SHOT-His-GUG, these two SHOT-RNAs were knocked down without affecting the corresponding mature tRNAs and cell growth was impaired compared with control (Shigematsu et al., 2017).

Some tsRNAs were recognized as microRNAs (miRNAs) at first (Venkatesh et al., 2016). Such mis-annotated tsRNAs play a miRNA-like role and correlate with BC. As reported, the expressions of tRF/miR-720, tRF/miR-1260, and tRF/miR-1280 were elevated in the blood of patients with ER-positive/HER2-negative BC (Huang et al., 2017). tRF/miR-720 is originated from tRNA-Thr, while tRF/miR-1260 and tRF/miR-1280 are processed from tRNA-Leu respectively (Park et al., 2014). Further studies will be valuable to define the clinical utility of tRF/miR-720, tRF/miR-1260, and tRF/miR-1280 levels in terms of treatment response.

##### 4.1.1.2 Ovarian cancer

Ovarian cancer is another sex hormone-associated cancer and one of the gynecologic malignancies with the highest mortality rate (Levine et al., 2016). Ovarian cancer is featured of nonspecific symptoms of early stage, leading to late diagnosis (Karlan et al., 2016).

It was reported that tRF-03357 promoted SK-OV-3 cell (an ovarian cancer derived cell line) proliferation, migration and invasion *via* downregulating its target gene Homeobox-containing protein 1 (HMBOX1) which was a transcription factor belonging to the hepatocyte nuclear factor family (Zhang et al., 2019a). HMBOX1 was found to accelerate cell apoptosis and inhibit cell proliferation (Yu et al., 2018). Its expression in ovarian cancer cells was significantly lower than that in ovarian epithelial tissues or normal ovarian epithelial cell lines (Yu et al., 2018). The analysis of the TCGA-OV dataset demonstrated that i-tRFs and high proportion of fragments derived from tRNA-Gly-GCC were abundant in ovarian tumors (Panoutsopoulou et al., 2021). Particularly, increased i-tRF-Gly-GCC levels were markedly correlated with worse survival and higher risk of disease progression in patients (Panoutsopoulou et al., 2021). Thus, these tsRNAs promote ovarian cancer.

Some tsRNAs inhibit ovarian cancer progression. For example, 5′ tRF-Glu bound to the 3′ UTR of breast cancer anti-estrogen resistance 3 (BCAR3) mRNA and decreased the expression level of BCAR3 which can suppress proliferation in ovarian cancer development (Zhou et al., 2017b). Furthermore, 5′-tRF-Glu can regulate apolipoprotein E receptor 2 (APOER2), indicating it has multiple targets (Deng et al., 2015). Hence, tsRNAs provide a potential diagnostic and therapeutic target for ovarian cancer.

##### 4.1.1.3 Prostate cancer

Prostate cancer (PCa) is the main leading cause of disease-related death among men globally (Sung et al., 2021). In the beginning, androgen-deprivation therapies and Platinum-based anticancer drugs (e. g., Cisplatin) were applied in PCa treatment; however, the two methods led to the resistance of cancer (Yang et al., 2021). Studies have raised the possibility that tsRNAs could function as potential indicators for cancer diagnosis and treatment in recent years. Several tsRNAs are proven to regulate the proliferation of PCa cells (Jia et al., 2020). As previously studied, tRF-1001 from a tRNA-Ser precursor was critical for cell proliferation and was highly expressed in the PCa cell lines (Olvedy et al., 2016). DNA synthesis, cell viability and cell proliferation could be inhibited when tRF-1001 was knocked down (Olvedy et al., 2016).

tRF-315, from tRNA-Lys-CTT, was highly expressed in PCa patients and the knockdown by siRNA targeting tRF-Lys-CTT could inhibit the proliferation of LNCaP cells (a PCa cell line) (Yang et al., 2021). Yang et al. (2021) found that cisplatin in PCa cells significantly increased the expression of tRF-315 which targeted the Gadd45a, a tumor suppressor gene, and regulated the cell cycle (Yang et al., 2021). In summary, tRF-315 can prevent cisplatin-induced apoptosis and alleviate cisplatin-induced mitochondrial dysfunction in LNCaP cells. In this way, tRF-315 can be regarded as a therapeutic target and a predictive indicator for cancer treatment.

Moreover, tRF-544 from tRNA-Phe-GAA was downregulated in PCa tissues compared to healthy counterparts (Olvedy et al., 2016). Therefore, the ratio of tRF-315/tRF-544 might be a potential clinical biomarker because the higher ratio represents poorer progression-free survival and shorter period to disease relapse (Yang et al., 2021).

##### 4.1.1.4 Chronic lymphocytic leukemia

Chronic lymphocytic leukemia (CLL) is a heterogeneous disease featured by the expansion of CD19^+^/CD5^+^ B cells and is the most common human adult leukemia (Lysák et al., 2015). CLL influences white blood cells and tends to progress slowly over many years. Usually, patients suffered from CLL are older than 60 (Sung et al., 2021).Ts-101, ts-53, ts-43, ts-44, ts-46, and ts-47 were pronouncedly downregulated in CLL samples (Balatti et al., 2022). Further studies should be carried out to determine the functions of tsRNAs in CLL.

##### 4.1.1.5 Lung cancer

Lung cancer is one of the most common cancers worldwide nowadays (Sung et al., 2021). At present, there are some investigations on the roles of tsRNAs in lung cancer, as well as the corresponding molecular mechanisms. The four tsRNAs, mentioned in CLL including ts-101, ts-53, ts-46 and ts-47, were also revealed to be downregulated in lung cancer (Balatti et al., 2017). Overexpression of ts-46 and ts-47 could significantly inhibit the colony formation of lung cancer cells, indicating that these tsRNAs had effects on lung cancer cell growth and survival (Balatti et al., 2027). On the contrary, the expression of tRF-Leu-CAG was higher in human non-small cell lung cancer (NSCLC) tissues than that in normal tissues, particularly in the late stage (Shao et al., 2017). The overexpression of tRNA-Leu-CAG derived tsRNA could promote cell proliferation and cell cycle G0/G1 progression and finally contributed to the promotion of NSCLC by targeting at Aurora kinase A (AURKA, Aurora A) (Damodaran et al., 2017). AURKA was essential in the progression of mitosis, centrosome maturation/separation and mitotic spindle function regulation (Tomlinson et al., 2022). In NSCLC, overexpression of AURKA was regarded as a poor prognosis and therefore AURKA inhibitors could not only cause G2/M arrest of cancer cells but also lead to the significant autophagy and enhance the anticancer activity of clinical medicine (Katayama and sen., 2010). In summary, knockdown of tRF-Leu-CAG by tRF-Leu-CAG inhibitor can repress AURKA, suppressing cancer cell proliferation and impeding cell cycle at last.

##### 4.1.1.6 Colorectal cancer

Colorectal cancer (CRC) ranks third of the most common malignance and is one main cause of cancer-related death in the world (Chen et al., 2019a). Although great efforts have been made to uncover the pathogenesis of CRC, the underlying mechanisms are still unknown to a large extend.

It was reported that tRF-20-M0NK5Y93 was downregulated in CRC cells under hypoxic conditions (Luan et al., 2021). The invasion and metastasis ability were enhanced when tRF-20-M0NK5Y93 was inhibited and EMT-related Claudin-1 was upregulated in cancer cells versus counterparts (Luan et al., 2021). Claudin-1 was a critical component in the tight junction protein family, distributing on the surface of the cell membrane and was correlated with tumor metastasis (Takasawa et al., 2021). In summary, tRF-20-M0NK5Y93 could inhibit CRC cell migration and invasion targeting Claudin-1during EMT. Likewise, when tRF-20-M0NK5Y93 was knocked down in RKO and SW480 cell lines (CRC-originated cell lines), the invasion and migration of these cells were promoted (Luan et al., 2021). Such results demonstrated that tRF-20-M0NK5Y93 might suppress the tumor progression and could act as a novel potential therapeutic target.

#### 4.1.2 Neurological disorders

Structural and functional disorders of nervous system induce neurological diseases (Faghihi et al., 2004). The expression of tsRNAs is detected to be significantly altered in the senescence-accelerated mouse prone 8 (SAMP8) model which is a model of Alzheimer’s and Parkinson’s diseases (Zhang et al., 2019b). For example, AS-tDR-011775, from tRF-1, was upregulated in SAMP8 model and was capable of promoting the disease progression by acting on Mobp gene (myelin-associated oligodendrocyte basic protein) and Park2 (Parkinson Disease Protein 2) (Zhang et al., 2019b). The dysregulated tsRNAs are associated with impaired synapse formation and disrupted synaptic vesicle cycle pathways (Zhang et al., 2019b).

Ischemic stroke is a kind of clinical disorder and leads to vascular and neuronal damage (Cerasuolo et al., 2022). Li et al. (2016) reported two tsRNAs from tRNA-Val-CAC and tRNA-Gly-GCC were overexpressed in multiple models including the rat brain ischemia model, the mouse hindlimb ischemia model, and the cellular hypoxia model, indicating that they might play crucial roles in ischemic pathophysiology and inhibit the proliferation, migration, and tube formation of endothelial cells (ECs) (Li et al., 2016). In conclusion, ischemic-hypoxic injuries induce the cleavage of specific tRNAs and these tsRNAs, regarded as signaling molecules, can modulate angiogenesis by regulating the function of ECs.

#### 4.1.3 Virus infection

Respiratory syncytial virus (RSV) is a leading cause of lower respiratory tract infection, especially in infants, the elderly, and immune compromised patients (Jo et al., 2021). Currently, there is no approved RSV vaccine and treatment worldwide (Williams et al., 2020). According to Choi et al. (2020)’s study, RSV infection induced several tRF-5s, which were derived from the 5′ end of a subset of tRNAs, such as 5′-tRF-Glu-CTC and 5′-tRF-Gln-CTG, regulating RSV replication and having gene *trans*-silencing function (Choi et al., 2020). Precisely, 5′-tRF-Glu-CTC targeted 3′ UTR of Apoer2 and promoted RSV replication (Choi et al., 2020). A mechanism-focus study revealed that RSV replication was modulating by suppressing the APOER2 which was a novel anti-RSV protein (Deng et al., 2015). What’s more, two specific RSV-induced tsRNAs including 5′-tRF-Gly-CCC and 5′-tRF-Cys-GCA were significantly increased in accordance with RSV infection, and promoted RSV replication synergistically (Zhou et al., 2017a). To control the RSV, inhibition of these tsRNAs with antisense treatment suppressed RSV proliferation and virus-associated inflammation (Zhou et al., 2017a).

Further, deep sequencing analysis indicated that tsRNAs were more abundant in human T-cell leukemia virus type 1 (HTLV-1) infected T cells compared with healthy ones (Ruggero et al., 2014). Among such tsRNAs, tRF-1001, being upregulated in PCa cells, was increased in HTLV-1-infected cells as well (Lee et al., 2009). In addition, tRF-3019 was reported to be upregulated in infected T cells and might be involved in HTLV-1 replication critically *via* functioning as a primer for HTLV-1 reverse transcriptase (Ruggero et al., 2014). Such investigations above suggest that tsRNAs may play a critical role in the medical treatment and management of viral infection.

#### 4.1.4 Other diseases

##### 4.1.4.1 Osteoporosis

Osteoporosis is a common orthopedic disease characterized by low bone mineral density (Fischer and Haffner., 2022). Little research has been studied on functions of tsRNAs in osteoporosis while the roles of messenger RNAs and miRNAs in osteoporosis have been comprehensively focused on. tRF-25 (tRF-25-R9ODMJ6B26), tRF-38 (tRF-38-QB1MK8YUBS68BFD2) and tRF-18 (tRF-18-BS68BFD2) could function as novel biomarkers with significant indication in diagnosing osteoporosis patients (Zhang et al., 2018). tRF-25, tRF-38 and tRF-18 had 25, 192 and 38 targeted genes respectively and were all upregulated in patients with osteoporosis compared to healthy people (Zhang et al., 2018). According to GO analysis, the targeted genes of differentially expressed tsRNAs were involved in transcription, axon guidance, neuron migration, cytoplasm, cell junction, protein binding and DNA binding (Cohen-Solal et al., 1993). These targeted genes were reported to participate in “Insulin signaling pathway,” “ErbB signaling pathway,” “PI3K-Akt signaling pathway,” “FoxO signaling pathway,” “Neurotrophin signaling pathway,” “cGMP-PKG signaling pathway,” “Wnt signaling pathway,” “MAPK signaling pathway,” “Ras signaling pathway,” “Calcium signaling pathway” and “Endocytosis” (Wu et al., 2017). Consistently, such signaling pathways including Wnt, PI3K-Akt, MAPK, TGF-beta and calcium were found to play critical roles in the development of osteoblasts (Greenblatt et al., 2013).

##### 4.1.4.2 Knee osteoarthritis

Osteoarthritis (OA) is a musculoskeletal disorder which has affected millions of people worldwide (Swain et al., 2022). It can ruin any joint, especially the knee, hip, spine and upper limb joints (Zacharjasz et al., 2021). As reported by United Nations, an estimated of 15% of the global populations who are over 60 will have symptomatic OA and one-third of these people will be eventually disabled, which means by 2050, 130 million people will suffer from OA globally and 40 million of them will be severely disabled (Korsh et al., 2016).

The knee is the largest synovial joint in humans, composed by osseous structures (distal femur, proximal tibia and patella), cartilage (meniscus and hyaline cartilage), ligaments and a synovial membrane (Younas et al., 2021). The knee is a frequent site for painful conditions due to weight bearing and being used extensively in daily lives (Younas et al., 2021). Green et al., studied the role of tRF-3003a, a type tRF-3 produced by the cleavage of tRNA-Cys-GCA, in post-transcriptional gene regulation in IL-1β stimulated chondrocytes (Greenblatt et al., 2013). The Janus Kinase and Signal Transducer and Activator of Transcription (JAK-STAT) kinase pathway regulates the expression of many cytokines, including IL-6, which plays an important role in the pathogenesis of multiple diseases like OA (Huang et al., 2007). In conclusion, tRF-3003a was capable of suppressing JAK3 kinase and inhibiting the progression of knee osteoarthritis, suggesting a potential role of tRF-3003a in the pathogenesis-based designing of novel therapies for OA.

The knowledge of tsRNAs in OA is so far sparse. It is surprising that in diverse tissues and organisms, tsRNAs are the most abundant group of small RNA next to miRNAs (Lee et al., 2009). Therefore, a wider exploration of the mechanism for expression and regulation of tsRNAs is of great value of OA diagnosis and prognosis.

Besides the tsRNAs mentioned above, some other kinds are also been suggested to have correlation with diseases. For example, tRNA-Ala-TGC-5-1 can be an indicator because it has more than 85% sensitivity and specificity in differentiating melanoma patients (Husna et al., 2022). The low expression of tRF-Pro-CGG betokens poor prognosis in pancreatic ductal adenocarcinoma (Li et al., 2021b). The circulated level of tRNA-Arg-CCT is impaired in patients with clear renal cell carcinoma compared to control (Zhao et al., 2018), likewise, in oral submucous fibrosis (OSF), tRF-Trp-CCA-007 is downregulated (Zeng et al., 2021). However, the function of tRF-Ile in regulating diseases is rarely reported.

### 4.2 Roles of tRNA halves in diseases

There are fewer studies on diseases regulated by tiRNAs compared to tRFs. Some studies have demonstrated that tiRNAs can be potential therapeutic targets for cancers. As Pekarsky et al. investigated, ts-3676 from tRNA-Thr and ts-4521 from tRNA-Ser were mutated and these two tiRNAs were significantly downregulated in CLL (Pekarsky et al., 2016). In addition, ts-3676 and ts-4521 could interact with Piwil2 and were found to be enriched in piwi-complex which could regulate DNA methylation (Veneziano et al., 2019). In breast cancer, 5′-tiRNA-Val was downregulated in patients and could inhibit Wnt/β-Catenin pathway by targeting FZD3 (Jia et al., 2020). Furthermore, tiRNA-5034-Glu-TTC-2 was reported to be downregulated in tissues of gastric cancer and patients with higher expression of tiRNA-5034-Glu-TTC-2 exhibited better curing effect compared to those with low expression (Zhu et al., 2019b). Functional analyses revealed that tiRNAs might regulate neurological disorders. Since tiRNAs were generated from tRNAs under stress and the levels of tiRNAs were strongly associated with the severity of cell damage, tiRNAs might function as the ideal biomarker of neuron damage (Elkordy et al., 2018). However, the accepted notion that tiRNAs are beneficial to cell survival under stress conditions is challenged recently. Sanadgol et al. (2022) had newly discovered a tRNA-Gly-derived tiRNA which could be induced by arsenite promoted the cell death suggesting the diverse functions of tiRNAs in cell viability (Sanadgol et al., 2022).tiRNAs, as novel non-coding RNAs, are garnering increasing focus in the field of study, but their functions and mechanisms remain largely unknown.

## 5 Mechanisms of tRNA-derived small RNAs in diseases

Among examples illustrated above, tsRNAs are abundant and display the potential to be biomarkers and therapeutic targets of diseases. The underlying mechanisms of tsRNAs are demonstrated below.

### 5.1 MicroRNA like regulation

miRNAs regulate gene expression by binding to complementary sequences at the 3′UTR end of the target mRNAs (Bartel, 2009). According to recent study, tsRNAs can behave like miRNAs and repress the expression of endogenous targets. To be more specific, tsRNAs have the ability to target the 3′UTR of certain mRNAs and repress their translation (Deng et al., 2015). For example, Deng et al. (2015) proposed that 3′ portion of a tRF-5, which was 5′-tRF-Glu-CTC, targeted the 3′UTR of Apoer2 (Deng et al., 2015). 5′-tRF-Glu-CTC was overexpressed upon RSV infection and promoted further infection by regulating the level of APOER2 (Deng et al., 2015).

In addition, tRF/miR-1280 could suppress CRC cell proliferation by inhibiting the Notch signaling pathway through directly interacting with the 3′ UTR of JAG2 mRNA (Huang et al., 2017). JAG2, a direct target of tRF/miR-1280, plays a role of a Delta-like Notch ligand which combines Notch 1 and Notch 2 receptors and promotes CRC metastasis through the regulation of cancer self-renewal (Wang et al., 2015). Notch signaling pathways control cell fate and signal integration in development and act important roles in regulating stem cell function as well (Liu et al., 2022). Likewise, tRF/miR-1280 was also found to be critical in pancreatic cancer by significantly decreasing JAG2 in pancreatic cancer derived cell lines such as HCT116, HCT15, and Panc-1 cells, at protein level (Huang et al., 2017).

**FIGURE 2:**
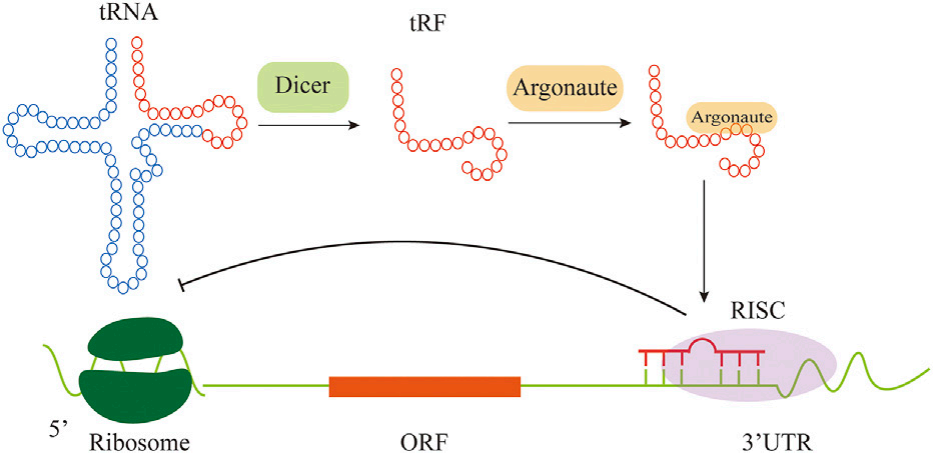
Some tRFs have miRNA-like function. The tRF is guided by AGO and is uploaded into RISC to regulate gene expression.

Another tRNA-derived fragment called CU1276, a 22-nt small RNA, is derived from tRF-Gly-GCC, and expressed in human mature B cells which was misunderstood as miRNA before because it functioned as a miRNA and repressed mRNA targets in an Argonaute (AGO)-dependent miRNA-like fashion (Maute et al., 2013). CU1276 is able to repress mRNA transcripts in a sequence-specific manner and also possesses the nature of a miRNA such as a DICER1-dependent biogenesis, and physical association with AGO proteins (Kuscu et al., 2018). To be specific, CU1276 repressed endogenous Rpa1 (Replication protein A) which was an important gene in many aspects of DNA dynamics including genome replication (Kuscu et al., 2018). The stable expression of CU1276 in Burkitt lymphoma-derived cell line led to an RPA1-dependent suppression of their proliferation (Haring et al., 2008). Taken together, the down-expression of CU1276 levels in lymphoma cell lines increased RPA1 protein level correspondingly, leading to a growth advantage to malignant cells (Maute et al., 2013). Besides, tsRNAs are capable of regulating gene expression through noncanonical miRNA-like actions in which tsRNAs bind with AGO proteins to form RNA induced silencing complex (RISC) (Figure 2) (Loss-Morais et al., 2013). AGO protein, as the core component of RISC, is made up of four domains: N terminus, PAZ, MID, and PIWI (Gu et al., 2021). Human AGO proteins consist of AGO1-4 which display ubiquitous tissue expression and PIWIL 1-4 which are mainly expressed in germline (Peters et al., 2007). As analyzed, tRF-5s and tRF-3s can associate with human AGO 1, 3 and 4, while few results show the association with AGO2 (Kumar et al., 2014).

### 5.2 tRNA-derived small RNAs work by binding to proteins

**FIGURE 3:**
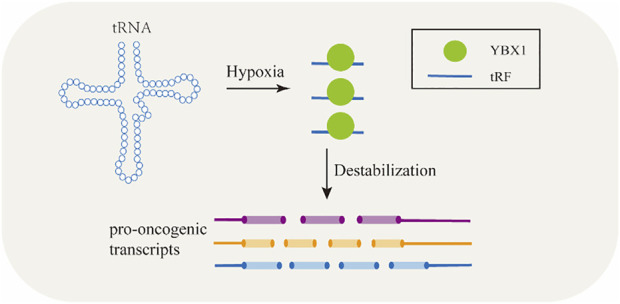
TRFs bind to RNA-binding proteins to regulate gene expression and modulate the target RNAs’ stability.

tsRNAs are capable of regulating gene expression by binding to proteins (Boskovic et al., 2020). Kumar et al. (2014) reported that certain tsRNAs preferred to combine with AGO proteins which played essential roles in gene silencing, e.g., tRNA-Glu-CTC bind with AGO1 and AGO4 to induce gene silencing (Kumar et al., 2014).Some tsRNAs regulate gene expression by binding to RBPs. There are approximately 1,500 RBPs in human genome (Foroushani et al., 2020). RBPs are a class of highly conserved proteins and are known to participate in post-transcriptional regulation and to serve multitude functions in RNA-driven processes such as splicing, polyadenylation, stability, transport, and translation by utilizing their RNA-binding domains (RBDs) (Castello et al., 2012). tsRNAs by binding to RBPs can regulate gene expression and control the targeted RNAs’ stability (Figure 3) (Krishna et al., 2019). YBX1 keeps transcripts stable including some oncogenes thus promote cancer cell proliferation by binding with endogenous oncogenic mRNA (Che et al., 2021). YBX1 can also combine with several kinds of regulatory RNAs, including tsRNAs (Wang et al., 2020c). Under hypoxic stress, the level of a subset of tsRNAs were elevated and in turn compete binding to YBX1 with oncogene transcripts which leads to mRNA degradation and inhibits proliferation of cancer cells eventually (Goodarzi et al., 2015). As reported in BC cells, under hypoxic conditions, several tsRNAs were induced to produce from tRNA-Asp, tRNA-Glu, tRNA-Gly, and tRNA-Tyr thus displaced the 3’ UTR of some oncogenic transcripts from the YBX1 protein, their stability was then suppressed (Goodarzi et al., 2015).

### 5.3 Regulation of translation

It is demonstrated that tsRNAs are capable of regulating translation in different manners such as affecting the biogenesis of protein through modulating the initiation of translation (Gebetsberger et al., 2017).

**FIGURE 4:**
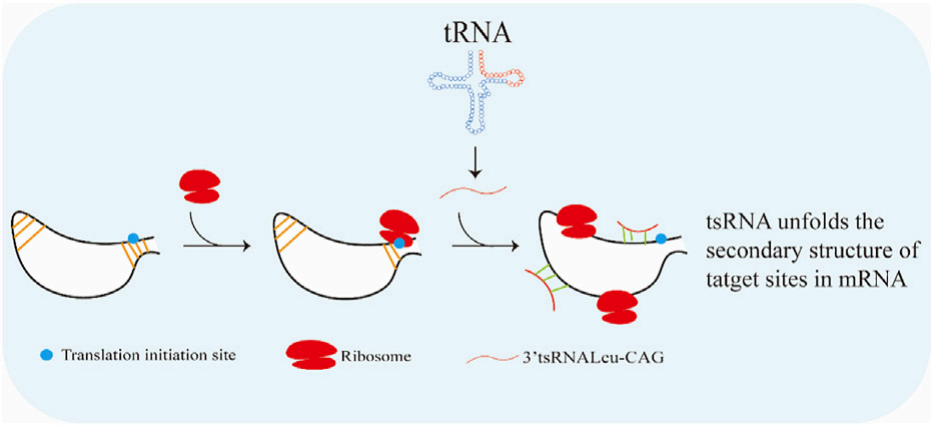
3′-tsRNA-Leu-CAG is able to increase translation in human cancer cells by binding to duplexed secondary target sites in RPS28 mRNA and unwound the hairpin secondary structure.

Promoting ribosome biogenesis can activate translation (Chan et al., 2011). Ribosomal protein S28 (RPS28) is necessary for 18S ribosomal RNA (rRNA) biogenesis and integration of the 40S ribosomal subunit (Malygin et al., 2010). The 3′ UTR target site of RPS28 mRNA constructs a secondary structure containing a translation initiation site (Kim et al., 2019). Translational activation features are found on tsRNAs (Li et al., 2021a). As reported, tsRNA-Leu-CAG (3′ end of Leu-CAG tRNA-derived fragments) was able to increase translation in human cancer cells by binding to duplexed secondary target sites in RPS28 mRNA and unwound the hairpin secondary structure (Figure 4) (Kim et al., 2019). In this way, 3′-tsRNA-Leu-CAG could promote cell growth and proliferation (Shao et al., 2017).

tiRNAs can directly inhibit protein synthesis resulting in the repression of translation (Anderson et al., 2011). The mechanism of tiRNAs inhibiting protein synthesis is not known, though their ability of inducing the assembly of stress granules (SGs) indicates their action on the translation initiation machinery (Emara et al., 2010). Yamasaki et al. (2009), reported that the transfection of natural 5′-tiRNAs, but not 3′-tiRNAs, inhibited global translation in U2OS cells (Yamasaki et al., 2009). In particular, 5′-tiRNA-Ala was potent translational repressor which interfered some functions of eIF4G, e.g., binding to mRNA or/and eIF4E (Anderson et al., 2011).

### 5.4 Response to cell stress

Continuous stress responses leading to inflammation and disease pathogenesis (Retta et al., 2016). When facing oxidative stress, the intracellular biogenesis of tsRNAs is repressed (Saikia et al., 2012). Under oxidative stress, it is reported that the activity of cytosine-5 RNA methyltransferase (NSUN2) can be repressed and tRNA methylation is reduced, finally resulting in the intracellular biogenesis of tsRNAs corresponding to the suppression of protein synthesis (Wu et al., 2021). It was also investigated that oxidative stress induced a direct conformational change in tRNA structure which promoted ANG cleavage of tRNA into tiRNA and the process happened much earlier than DNA damage (Elkordy et al., 2019).

AlkB homolog 1 (ALKBH1), as a tRNA demethylase catalyzes demethylation of m^1^A in tRNAs and contain a tRNA-binding motif (Liu et al., 2016). ALKBH1-mediated tRNA demethylation can impact tRNA stability and increase cleavage (Elkordy et al., 2019). For example, tRNA-Glu-CTC, the Alkbh1 substrate, was cleaved into 2 distinct 5′tiRNA fragments (Rashad et al., 2020).

**FIGURE 5:**
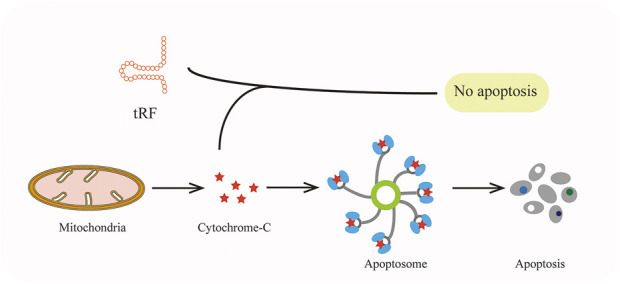
TRFs can bind to cytochrome-C to prevent apoptosis.

Demethylase α-KG-dependent alkB homolog3 (ALKBH3) could catalyze the demethylation which made tRNA more sensitive to the cleavage of ANG and generate 5′-tRF-Gly-GCC with ease (Chen et al., 2019b). Such tsRNAs interact with Cytochrome C (Cyt c) which is released from the mitochondria and then facilitate ribosome assembly and eventually suppress apoptosis of cervical cancer cells (Figure 5) (Saikia et al., 2014).

### 5.5 Regulation of kinase activity

tsRNAs have been found to regulate kinase activity. As Shao et al. (2017) reported, tRF-Leu-CAG regulated cell proliferation and cell cycle progression in NSCLC (Shao et al., 2017). The expression of AURKA was inhibited when tRF-Leu-CAG was knocked down (Shao et al., 2017). In other words, overexpression of tRF-Leu-CAG increased the activity of AURKA and thus promoted cell cycle progression at the G0/G1 phase in NSCLC, which indicated that tsRNAs like tRF-Leu-CAG could regulate cell cycle progression by modulating AURKA activity (Braicu et al., 2019).

## 6 Conclusion and perspectives

Studies on tsRNAs have opened a new era of their potential uses: in the diagnosis and prognosis of multiple diseases. We summarized the functions of diverse tsRNAs in different diseases and the underlying mechanisms. This review can provide valuable cues for further research of tsRNAs. Besides the functional tsRNAs in the above-mentioned diseases, there are also other types of disease related tsRNAs, but the amount and depth of these investigation are still in their infancy, in the meantime, the future study is urgently needed.

Meanwhile, in the pursuit of tRNA-based therapeutics, besides tsRNAs, many researchers focus on designing tRNAs to bypass premature stop signals and incorporate desired amino acids instead (Dolgin, 2022). Approximately 11% of inherited diseases are resulted from nonsense mutations, thus a suppressor tRNA is needed to eliminate premature termination of translation (Wang et al., 2022c). Back to 1982, it was reported that construction of a functional suppressor tRNA gene can be an approach to gene therapy for *β*-thalassaemia (Temple et al., 1982).

When compared with other gene therapeutic agents, tRNA therapy is of obvious advantages because tRNA is universal in treating multiple diseases. tRNA therapy has raised much attention and many breakthroughs have been made in recent years. In the future, we will continuously focus on the functions and mechanisms of tsRNAs and other tRNA-based therapeutics.
